# Surgical microscope with integrated fluorescence lifetime imaging for 5-aminolevulinic acid fluorescence-guided neurosurgery

**DOI:** 10.1117/1.JBO.25.7.071202

**Published:** 2020-02-24

**Authors:** Mikael T. Erkkilä, David Reichert, Nancy Hecker-Denschlag, Marco Wilzbach, Christoph Hauger, Rainer A. Leitgeb, Johanna Gesperger, Barbara Kiesel, Thomas Roetzer, Georg Widhalm, Wolfgang Drexler, Angelika Unterhuber, Marco Andreana

**Affiliations:** a Medical University of Vienna, Center for Medical Physics and Biomedical Engineering, Vienna, Austria; b Medical University of Vienna, Christian Doppler Laboratory OPTRAMED, Vienna, Austria; c Carl Zeiss Meditec AG, Advanced Development Microsurgery, Oberkochen, Germany; d General Hospital and Medical University of Vienna, Institute of Neurology, Vienna, Austria; e General Hospital and Medical University of Vienna, Department of Neurosurgery, Vienna, Austria

**Keywords:** fluorescence-guided neurosurgery, fluorescence lifetime imaging, protoporphyrin IX, surgical microscope

## Abstract

**Significance:** 5-Aminolevulinic acid (5-ALA)-based fluorescence guidance in conventional neurosurgical microscopes is limited to strongly fluorescent tumor tissue. Therefore, more sensitive, intrasurgical 5-ALA fluorescence visualization is needed.

**Aim:** Macroscopic fluorescence lifetime imaging (FLIM) was performed *ex vivo* on 5-ALA-labeled human glioma tissue through a surgical microscope to evaluate its feasibility and to compare it to fluorescence intensity imaging.

**Approach:** Frequency-domain FLIM was integrated into a surgical microscope, which enabled parallel wide-field white-light and fluorescence imaging. We first characterized our system and performed imaging of two samples of suspected low-grade glioma, which were compared to histopathology.

**Results:** Our imaging system enabled macroscopic FLIM of a $6.5\times 6.5\;{\mathrm{mm}}^{2}$ field of view at spatial resolutions <20  μm. A frame of 512×512  pixels with a lifetime accuracy <1  ns was obtained in 65 s. Compared to conventional fluorescence imaging, FLIM considerably highlighted areas with weak 5-ALA fluorescence, which was in good agreement with histopathology.

**Conclusions:** Integration of macroscopic FLIM into a surgical microscope is feasible and a promising method for improved tumor delineation.

## 1 Introduction

In brain tumor therapy, the key factor for improved patient outcome is complete resection.^1^ Even though fluorescence-guided surgery using 5-aminolevulinic acid (5-ALA)-induced protoporphyrin IX (PpIX) allows the surgeon to locate malignant tissue, its use is mostly limited to high-grade tumors.^2^ As a consequence, current research has focused on more sensitive detection schemes to visualize fluorescence that is not visible to the surgeon’s eye. However, most of the proposed *in vivo* methods rely on endoscopic platforms with limited field of view (FOV).^3^ Hence, visualization methods with increased FOV and working distance (WD) are needed to inspect larger areas. More sensitive detection of PpIX fluorescence is limited among others by tissue autofluorescence. Therefore, spectrally resolved fluorescence imaging^3^ has been proposed to detect nonvisible PpIX accumulations. However, it relies on the knowledge or parallel measurement of the optical tissue properties. This requires additional measurement channels making these devices more complex. On the other hand, fluorescence lifetime imaging (FLIM) relies on the time delay between the excitation and subsequent fluorescence emission and is thereby intrinsically independent of any intensity variations due to altered scattering or absorption in the tissue.^4^

To enable surgeons to use FLIM methods in a clinically familiar manner, we developed a frequency-domain and raster-scanning FLIM system incorporated into a commercial surgical microscope. We describe the overall architecture of our device and characterize the system in regard to future intraoperative use. Finally, we validate our method by imaging human brain tumor samples and compare the results to conventional PpIX fluorescence imaging as well as histology.

## 2 Methods

Based on the feedback from neurosurgeons, we opted for a minimum WD of 200 mm. We modified the assistant’s surgeon port of the surgical microscope (OPMI Visu 200, Carl Zeiss Meditec, Germany) for integration of the FLIM system and designed a compact scanner block, where we integrated the fiber port for the excitation laser as well as the beam steering and collimation optics. The object-sided numerical aperture (NA) computed using Code V was 0.013, implying a theoretical diffraction-limited resolution of 19  μm. Due to the low NA, we attached the detector directly to one of the camera ports of the surgical microscope instead of coupling the fluorescence light through the scanners back into a fiber. This nondescanned detection scheme is often found in multiphoton microscopy.^5^

**Fig. 1:**
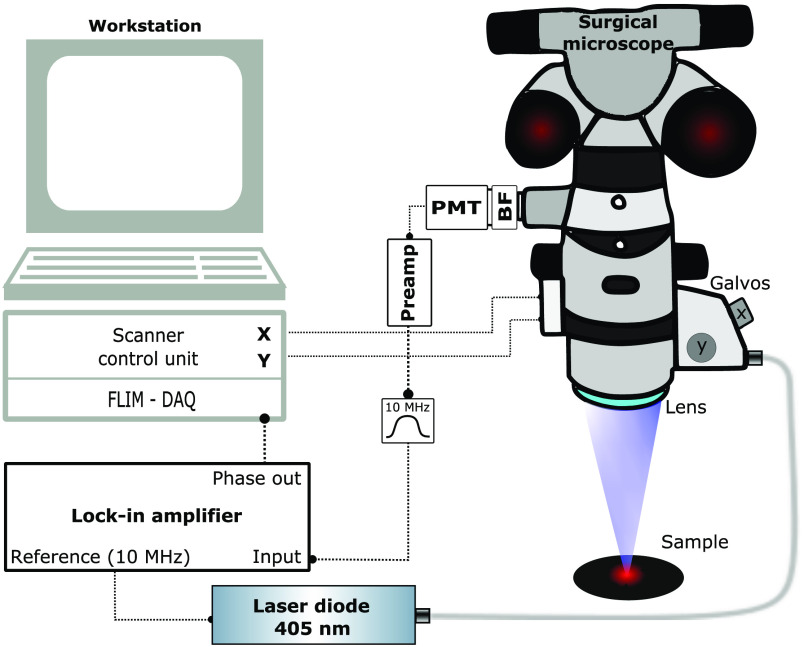
Sketch of FLIM surgical microscope. BF, bandpass filter; DAQ, data acquisition card; PMT, photomultiplier. A figure of the modified microscope head is provided in Supplemental Fig. S1.

In brief, the FLIM system is based upon laser scanning an amplitude-modulated 405-nm laser (phoxX-405, Omicron Laserage, Germany) for excitation and performing a nondescanned homodyne detection using a sensitive photomultiplier (PMT) as detector (H11901-20, Hamamatsu, Japan). As shown in Fig. 1, a lock-in amplifier (HF2LI, Zurich Instruments, Switzerland) generated a 10-MHz sine wave, which was used as analog modulation input for the laser. The laser was coupled into the scanner block and the galvanometer scanners (Saturn 1B, ScannerMax, Orlando, Florida) steered the beam across the sample covering a squared FOV of $6.5\times 6.5\;{\mathrm{mm}}^{2}$. Sampling of the field was performed with 512×512  pixels. This corresponds to a step size of 9  μm, which is close to the step size required for Nyquist limited sampling. Although the FOV could be enlarged to up to $20\times 20\;{\mathrm{mm}}^{2}$, the larger FOV would require longer acquisition times of around 10 min when sampling with the same step size. The incident laser power on the sample was 3.9 mW. Excited fluorescence was blocked by a 590- to 740-nm bandpass filter (665/150 BrightLine HC, Semrock, Rochester, New York) and detected by a PMT. The signal was then preamplified (C6438-01, Hamamatsu, Japan) and filtered by a 10-MHz bandpass (BBP-10.7+, Mini-Circuits, Brooklyn, New York) before detection by a lock-in amplifier. Using this homodyne detection scheme, the periodic signal was demodulated with the excitation wave as reference to obtain the modulation depth as well as the relative phase shift. Prior to any measurement, the system was referenced using a glass cuvette filled with allura red (CAS No. 25956-17-6, Sigma-Aldrich, St. Louis, Missouri) diluted in water to 5 mM. This food dye has a very short fluorescence lifetime of around 10 ps.^6^ Hence, it was used to compensate for the intrinsic time-of-flight path length of our system and to set the initial phase to 0.

For comparative wide-field imaging, a consumer grade color camera (EOS 5D Mark II, Canon, Japan) was attached to the other camera port. Two camera settings were used. First, an image under white-light illumination was acquired. Subsequently, the white-light illumination was switched off and the 405-nm laser was scanned over the sample repeatedly. During the laser scan, the camera was set to a long exposure (10 s). The pixel dwell time was set such that the laser was scanned over the full FOV four times during the exposure time of the camera. A 430-nm longpass filter (FF01-430/LP-25, Semrock, Rochester, New York) was used to block the excitation laser and detect the fluorescence only. The integrated color fluorescence image was then overlaid on the white-light image in postprocessing.

The fluorescence lifetime acquisition was controlled through an open-source microscopy suite (ScanImage, Vidrio Technologies, Ashburn, Virginia^7^) in which we implemented an on-the-fly processing routine. This included the conversion of the measured voltage into the phase and computing the lifetime τ according to

$$\tau =-\frac{1}{2\pi f}\cdot \tan\;\Phi ,$$ (1)

where f is the modulation frequency and Φ is the measured phase. The processing and visualization required (8.3±0.8) ms on average for a 512×512  pixels frame.

## 3 Results and Discussion

**Fig. 2:**
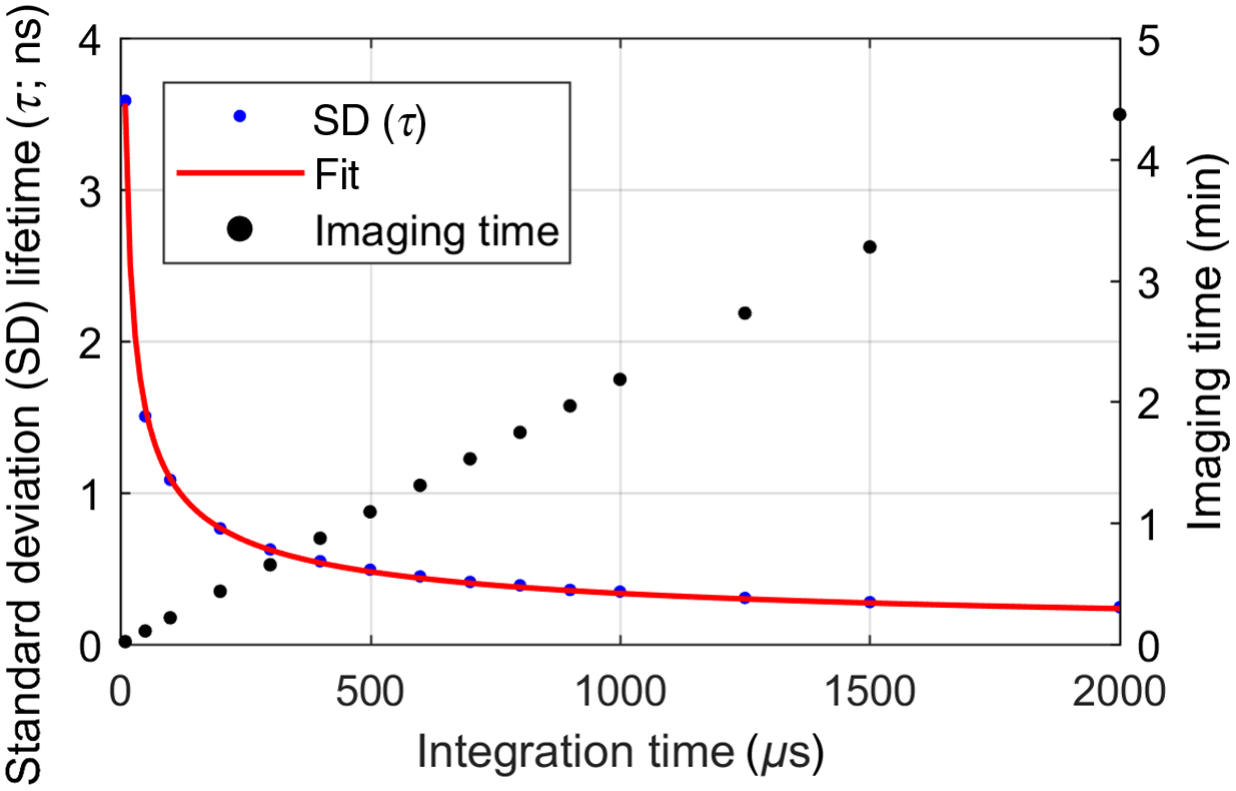
Fluorescence lifetime accuracy (dye: allura red) and imaging time (512×512  pixels) as a function of the lock-in amplifier integration time. The pixel dwell time was set to half of the integration time.

In the first step, we characterized the lifetime accuracy for integration times of the lock-in amplifier ranging from 10  μs to 2 ms. We first referenced the system using allura red with 10-ms integration time and set the initial phase and lifetime to 0. As shown in Fig. 2, higher time constants led to an increased lifetime accuracy, which followed a square root-like behavior (see fitted curve). When scanning, we found a pixel dwell time of half-the-time constant to be the best compromise between a fast acquisition and the settling of the lock-in amplifiers output. In this case, the step response of the lock-in amplifier reaches ∼1/e (36.8%). This was found to be sufficient, as intensity differences between adjacent autofluorescent and higher fluorescent areas in our samples were in the range of 60% to 70%. The corresponding acquisition times are plotted in Fig. 2. Although higher integration times improved the accuracy, it came at the cost of increased acquisition time. Therefore, we found the best compromise at a time constant of 500  μs where the lifetime showed <500  ps standard deviation and a single frame took ∼65  s. To validate our observations, we measured the fluorescence lifetime of three selected fluorescent dyes. Fluorescein (CAS No. 2321-07-5, Sigma-Aldrich) was dissolved in phosphate-buffered saline and diluted to a concentration of 4  μM. The pH was measured to be 7.0. Furthermore, coumarin 153 (C153, CAS No. 53518-18-6, Sigma-Aldrich) was dissolved in ethanol absolute (CAS No. 64-17-5, VWR International, Fontenay-sous-Bois, France) with a concentration of 10  μM. For PpIX, a custom-made reference sample embedded in polymethylmethacrylate (Starna Scientific Ltd., Ilford, United Kingdom) was measured.^8^ The measured lifetimes agreed within the range of error margins with reported literature values for the dyes in the respective solvent (Table 1). The strongest measured lifetime deviation respective to literature (0.2 ns) was measured for fluorescein, which could be caused by the pH dependence of fluorescein lifetime.^9^ Lifetime standard deviations were computed according to the law of error propagation as a sum of the errors induced through the referencing process and the actual lifetime measurement. Furthermore, the lateral resolution was determined using a positive fluorescent USAF 1951 target and oversampling the FOV. The second element of the fifth group (35.9  line pairs/mm) was still resolvable with >20% contrast for the fast and slow scanning axes (Supplemental Fig. S2). This is in good agreement with the theoretically expected resolution of 19  μm.

**Table 1:** Fluorescence lifetimes ($\tau_{\mathrm{mea.}}$) measured for selected fluorophores. $\tau_{\mathrm{rep.}}$: reported literature values.

Sample	$\tau_{\mathrm{mea.}}$ (ns)	$\tau_{\mathrm{rep.}}$ (ns)
Fluorescein	3.8±0.2	4.0^9^
Coumarin 153	4.9±0.2	4.8^10^
${\mathrm{PpIX}}_{\mathrm{Ref}.\text{sample}}$	5.5±0.3	5.5±0.2^8^

**Fig. 3:**
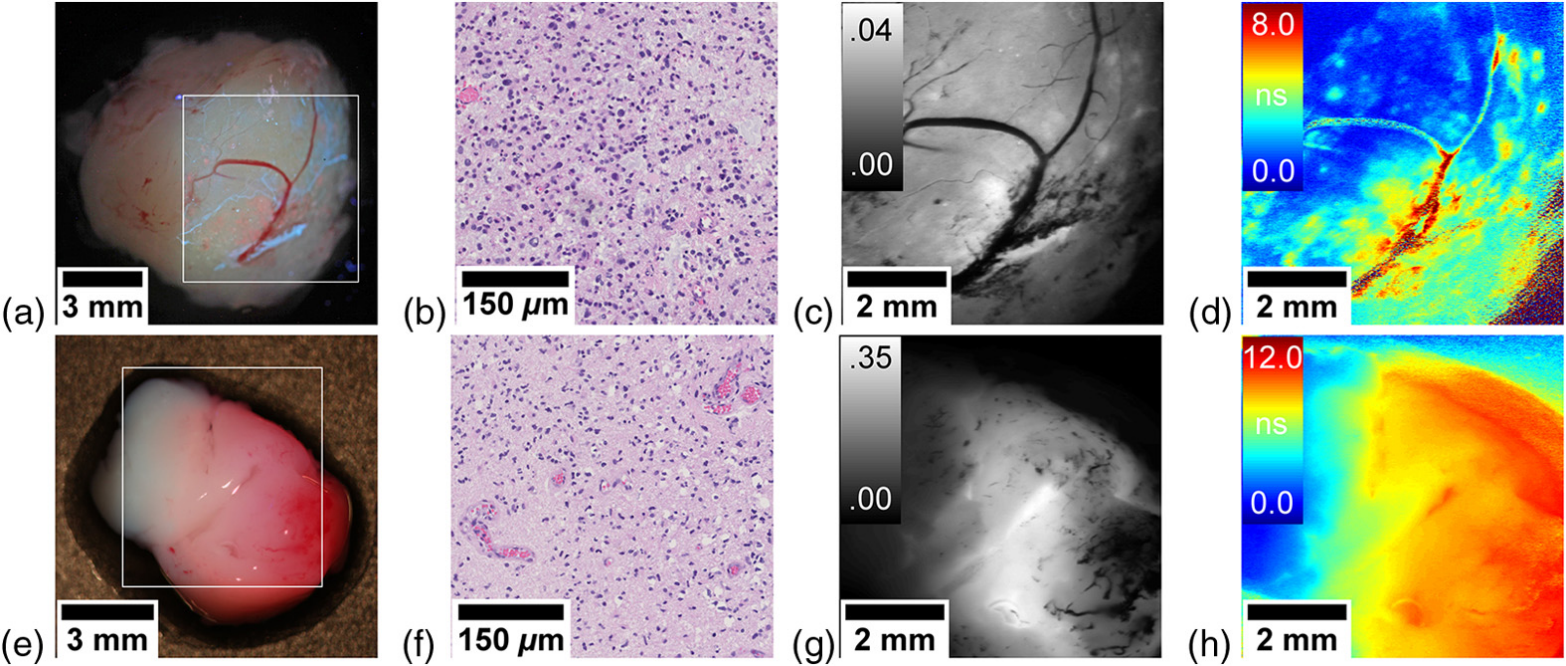
Brain tumor samples. (a)–(d) WHO grade III astrocytoma and (e)–(h) WHO grade III oligodendroglioma. Conventional white-light image by a consumer grade camera with fluorescence overlay. (a), (e) The white rectangle indicates the area of the scan; (b), (f) representative histopathological section; (c), (g) fluorescence intensity (rel.) acquired with the PMT; and (d), (h) fluorescence lifetime map (ns).

Imaging with the FLIM system was performed *ex vivo* on human tumor samples within an ongoing study approved by the ethics committee of the Medical University of Vienna (EK419/2008—Amendment 04/2018). We show two representative samples from two patients undergoing tumor resection of suspected low-grade gliomas after obtaining informed and written consent. A detailed study design can be found in our previous publication.^8^ Tissue samples were imaged within an hour after resection and kept moist using artificial cerebrospinal fluid. For the first patient, the surgeon did not report any visible PpIX fluorescence. However, when we imaged the sample, we observed very weak red spots as well as blue streaks in the wide-field fluorescence image as shown in Fig. 3(a). Note that the camera captured all fluorescence >430  nm. This led to the additional detection of blue fluorescence, which could be induced by nicotinamide adenine dinucleotide or other endogeneous fluorophores. However, in the following, we concentrated on measuring the PpIX fluorescence only as our system was conceived with a 590- to 740-nm bandpass filter in front of the PMT. Although one can identify several hot spots of higher brightness in the fluorescence intensity image in Fig. 3(c), FLIM offers a much better contrast [Fig. 3(d)] and identifies accumulations of PpIX with lifetimes up to 8 ns, which are neither visible in the laser scanning nor the wide-field fluorescence intensity images. These high fluorescence lifetimes suggested the presence of a high-grade focus. Indeed, in the diagnostic work-up, the tumor was confirmed to be a focally anaplastic isocitrate dehydrogenase (IDH)-mutant astrocytoma (WHO grade III) with marked cellular pleomorphism, increased proliferative activity, and myxoid-cystic degeneration [Fig. 3(b)].

In the second patient, the surgeon excised a focal fluorescent area, which was then further characterized using FLIM. Although the fluorescence was clearly visible in the camera image [Fig. 3(e)] and exhibited an increased fluorescence intensity [Fig. 3(g)], the fluorescence lifetime map [Fig. 3(h)] was more specific on the infiltrative tumor borders in regard to the adjacent physiological parenchyma, where fluorescence lifetimes <2  ns are expected due to tissue autofluorescence.^11^ Contrarily, regions with high lifetimes of up to 12 ns were indicative for a high-grade tumor. This suspicion was confirmed by the diagnostic work-up, in which the tumor was classified as IDH-mutant and 1p/19q-codeleted anaplastic oligodendroglioma (WHO grade III) with partly clear-celled appearance, brisk mitotic activity, and early vascular proliferations [Fig. 3(f)]. Note that the upper right corner of the lifetime map [Fig. 3(h)] shows increased lifetimes in an area that lies outside of the sample. Here, the artificial cerebrospinal fluid surrounding the sample diffusely scattered the laser beam into the tissue, thus exciting the highly fluorescent sample. As the PMT records the whole FOV, this fluorescence is also detected.

Our first results seem promising for enhanced tumor detection. Both specimen imaged with our system showed areas with lifetimes <2  ns, which corresponded well to the range of reported autofluorescence lifetimes of tumor-adjacent physiological brain parenchyma.^11^ Increased lifetimes clearly indicated the accumulation of PpIX,^8^^,^^11^ which enabled more sensitive tumor delineation than fluorescence intensity. This was supported by the fact that both tissue samples either had blood vessels or hemorrhages. When blood is covering tissue, it hinders the excitation of PpIX by absorbing the excitation light. Hence, the fluorescence intensity was lower in these areas. In contrast, FLIM of PpIX enabled imaging of those regions with only minor loss in contrast and therefore seemed to be less affected by the blood absorption. However, caution in interpreting those areas has to be taken, as even lower signals can lead to a higher lifetime variability, which could be confused with increased lifetimes. A metric for future application could be to highlight regions with increased lifetime variability to avoid false conclusions. Nonetheless, this is remarkable as the only alternative solution we are aware of is to avoid blood absorption using excitation of PpIX at 633 nm,^12^ where the fluorescence yield is ∼50 times lower.

At present, the acquisition time of around a minute limits the potential for real-time surgical guidance. Future studies should focus on increasing the imaging speed. Due to the stereoscopic design and the long WD, the NA of commercial surgical microscopes is currently limited to ∼0.02. This could be improved by introducing a beam splitter before the stereo path, thus making use of the full opening angle of the microscope objective. Another option would be to operate the system with a reduced integration time as well as with more sparse sampling. Fluorescence lifetime maps with reduced spatial and temporal resolution could still be of use to the surgeon when overlaid on the white-light image of the surgical field. In addition, increasing the laser power would allow for faster acquisition. While the power on the sample in this work was 3.9 mW, the maximum permissible exposure for skin according to ANSI Z136.1 permits 19.2 mW. However, our current laser throughput is limited by the reduced reflectivity of standard silver coatings found on the scanner mirrors. In future, we plan to upgrade our system to mirrors optimized for 405 nm. It should also be noted that this study has been performed such that images were acquired in the laboratory under low-light conditions. Additional studies are needed to evaluate the performance with the ambient light present in a surgical theater.

## 4 Conclusion

We presented the characterization and validation of a modified surgical microscope with integrated FLIM capability. While surgical microscopes have been equipped with alternative modalities such as spectroscopic imaging,^3^ to our knowledge, we are the first to integrate FLIM of PpIX. In contrast to conventional intensity imaging, the lifetime contrast allows to visualize pathological areas, which would otherwise be invisible. The integration of the technology into a surgical microscope favors the potential translation toward clinical applications.
