# Supplementary figures and images for: Surgical microscope with integrated fluorescence lifetime imaging for 5-aminolevulinic acid fluorescence-guided neurosurgery
Source: J Biomed Opt. 2020 Feb 24;25(7):071202. doi: 10.1117/1.JBO.25.7.071202 (PMC7039165; doi:10.1117/1.JBO.25.7.071202)

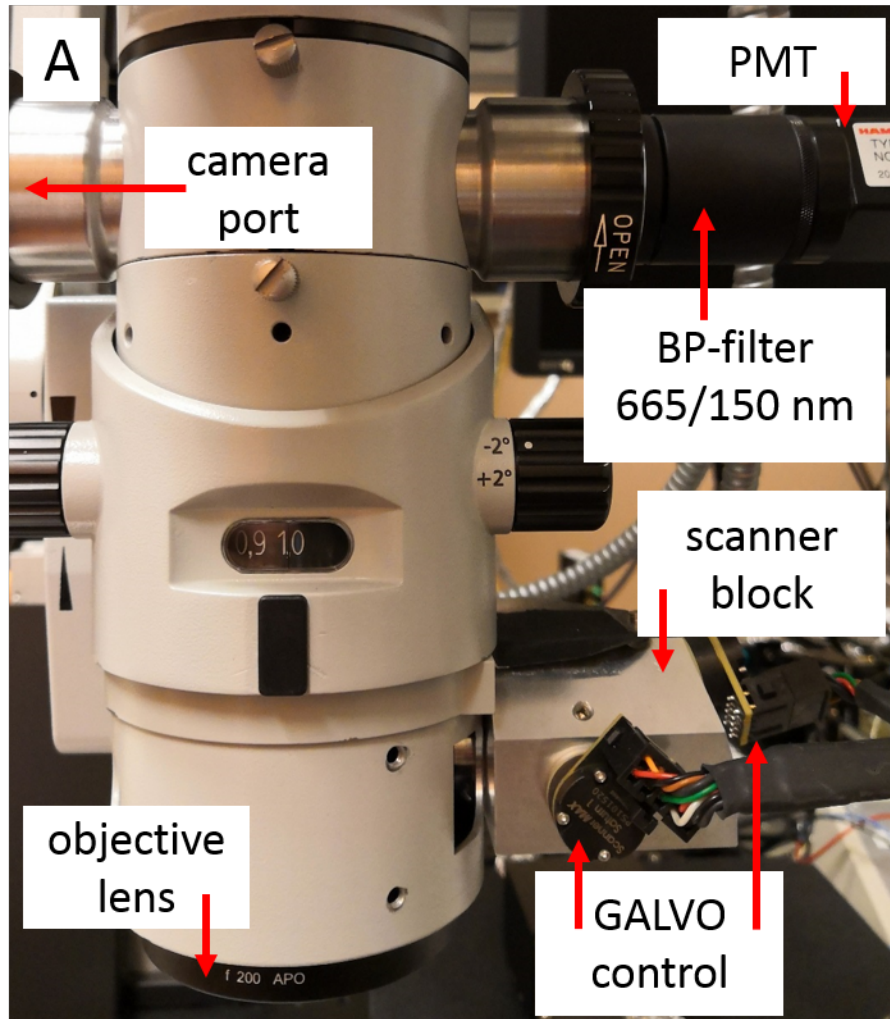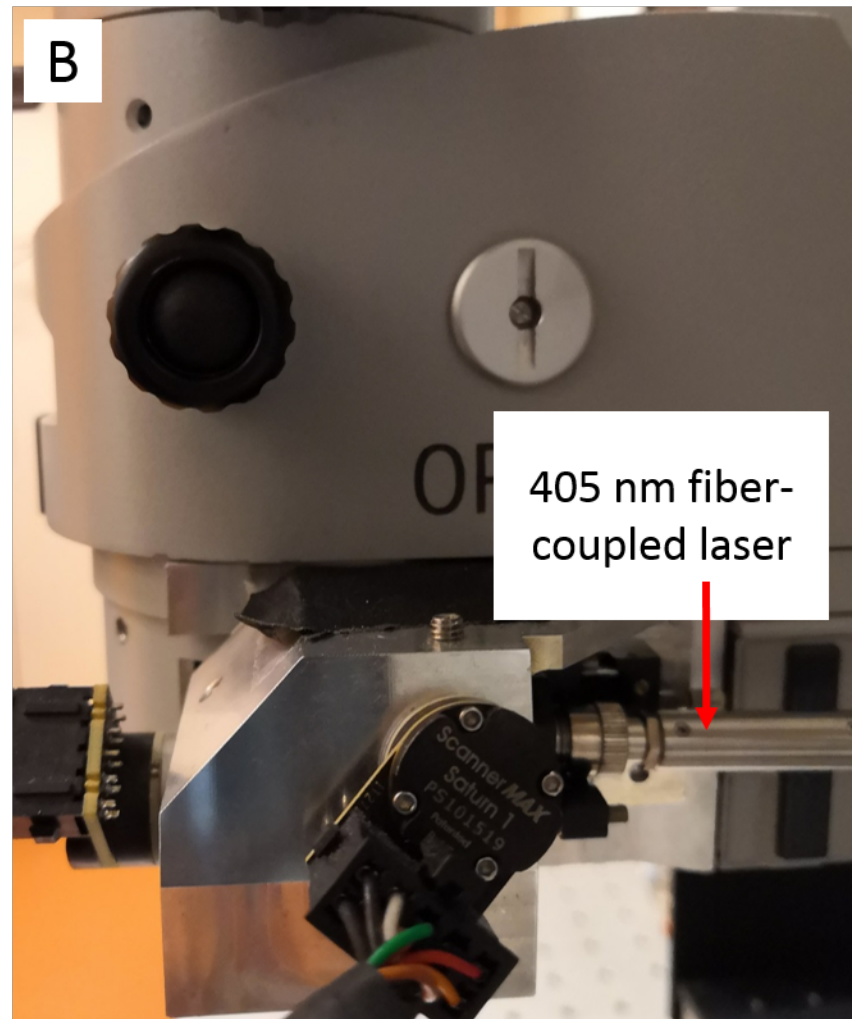

Supplement: Supplementary file 1 [file JBO_025_071202_SD001.pdf]

A

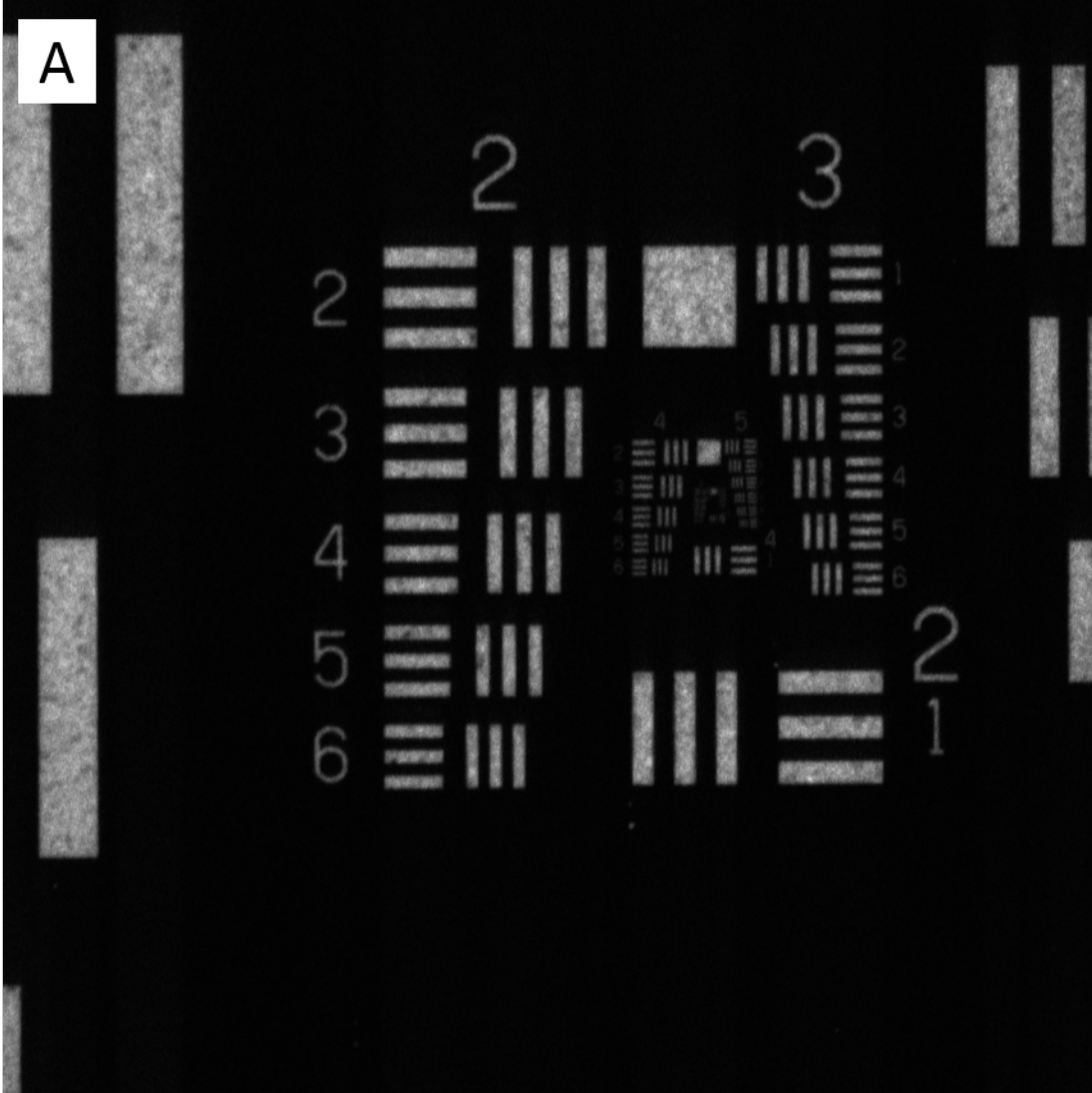

B

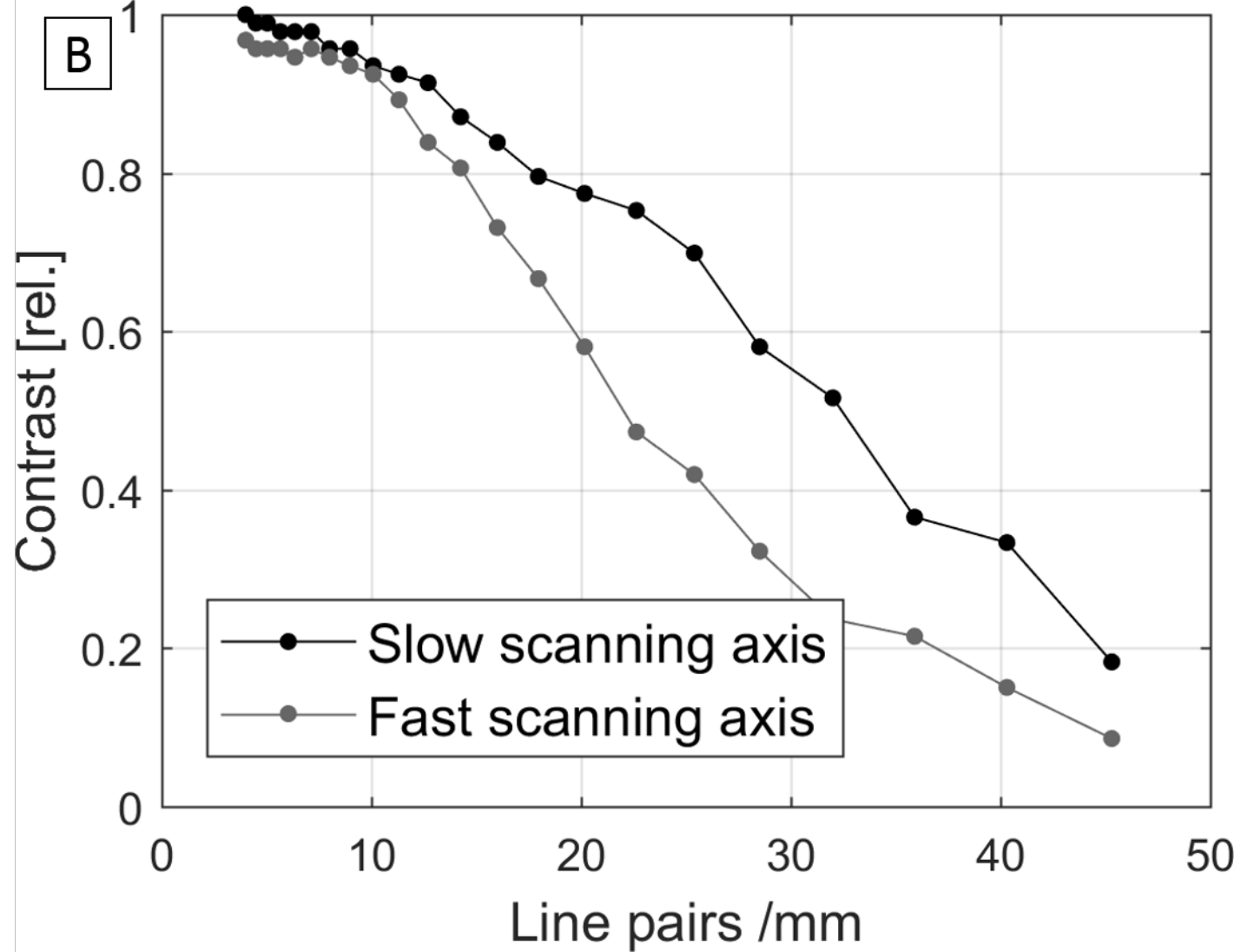

Supplement: Supplementary file 2 [file JBO_025_071202_SD002.pdf]
